# Dynamics of oscillators globally coupled via two mean fields

**DOI:** 10.1038/s41598-017-02283-1

**Published:** 2017-05-18

**Authors:** Xiyun Zhang, Arkady Pikovsky, Zonghua Liu

**Affiliations:** 1 Department of Physics, East China Normal University, Shanghai, 200062 P. R. China; 2 Institute for Physics and Astronomy, University of Potsdam, Karl-Liebknecht-Str. 24/25, 14476 Potsdam-Golm, Germany; 3 Department of Control Theory, Nizhny Novgorod State University, Gagarin Av. 23, 606950 Nizhny Novgorod, Russia

## Abstract

Many studies of synchronization properties of coupled oscillators, based on the classical Kuramoto approach, focus on ensembles coupled via a mean field. Here we introduce a setup of Kuramoto-type phase oscillators coupled via two mean fields. We derive stability properties of the incoherent state and find traveling wave solutions with different locking patterns; stability properties of these waves are found numerically. Mostly nontrivial states appear when the two fields compete, i.e. one tends to synchronize oscillators while the other one desynchronizes them. Here we identify normal branches which bifurcate from the incoherent state in a usual way, and anomalous branches, appearance of which cannot be described as a bifurcation. Furthermore, hybrid branches combining properties of both are described. In the situations where no stable traveling wave exists, modulated quasiperiodic in time dynamics is observed. Our results indicate that a competition between two coupling channels can lead to a complex system behavior, providing a potential generalized framework for understanding of complex phenomena in natural oscillatory systems.

## Introduction

Dynamics of globally coupled oscillators attracted large attention recently. It is relevant for many physical systems, like Josephson junctions, lasers, arrays of spin-torque and electronic oscillators^1–4^, but also for many life and social systems^5–7^. The paradigmatic model in this field is the Kuramoto-Sakaguchi model of globally coupled phase oscillators, describing a transition to synchronization if the attractive coupling is strong enough to overcome the natural spreading of oscillators frequencies^8, 9^. The global coupling typically appears in two setups. In one situation there are many links mutually connecting the oscillators in the population, so that the all-to-all coupling is a suitable description. Such systems are widely considered in neuroscience, where neurons are connected by an enormous number of synapses. In physical applications, in many cases the second setup is relevant, where the global coupling is due to a “global mode” which is fed by the units and acts back on them. For example, for Josephson junctions, electronic and spin-torque oscillators, the global coupling is due to a global current^3, 4, 10^ which flows through the units in series; for lasers the coupling is due to a global optical mode^2^; for metronomes, pendulum clocks, and for pedestrians on a bridge this global mode is the oscillation mode of the support^7, 11^. Note that the global signal can be rather complex and, in particular, include several harmonics of the basic frequency^12^. For example, horizontal oscillations of the support lead to a standard Kuramoto-Sakaguchi coupling of pendulum clocks via the first harmonics, while vertical oscillations produce a second-harmonics coupling^13, 14^. We nevertheless will dub this situation as one-mean-field (one-channel) global coupling, as there is only one mediator for the global mode.

Global coupling can also differently force different oscillators, e.g., if the global optical mode is oblique, an array of lasers will driven with different phase shifts. Another example is the coupling via an acoustic/electronic receiver-emitter scheme^15^, where the phase shifts experienced by oscillators depend on the propagation time of the global signal from emitter.

In this paper we generalize the Kuramoto coupling scheme and study the dynamics of oscillators driven by *two mean fields* via two different channels. A possible setup could be electronic/acoustic oscillators, two mean fields of which are collected by two receivers that drive the oscillators via two emitters. In optics, the two-channel coupling can be accomplished via splitting the global light mode beam and feeding it back with different phase shifts for the two parts. In life systems, in particular in coupled oscillating cells^6^, two global coupling channels can be realized with two different messengers that carry signals from the cells into the mixing environment, with possibly two different chemical mechanisms of influencing oscillations in the cells. For example, in neuronal system, the signals between neurons can be transferred by both chemical transmitters and electrical coupling channels. So the neurons are actually coupled by two mean fields. Interaction through different channels is also characteristic for physiological problems, where, e.g., cardiac and respiratory systems show coexisting couplings^16–19^. Another situation with two mean-field couplings, is related to the attempts to control synchrony in a population of coupled oscillators^20, 21^. Such an approach has been e.g. discussed in the context of suppression of collective brain oscillations at Parkinson’s disease^22–24^. Here the two channels of coupling are the internal (uncontrollable) one, and the external coupling due to an imposed feedback. Both couplings can be considered to a good approximation as global ones, acting on the whole population of the involved neurons.

Potentially, the most simple experimental realization of the two mean field coupling would be an extension of a recent experimental setup where Kuramoto-Sakaguchi coupling scheme with linear and nonlinear couplings have been experimentally verified^25^. The scheme for the two-field coupling is presented in Fig. 1 of the supplementary material. Instead of one common resistive load, like in the experiment^25^, one could implement two such loads, and additionally modify the phase shifts of actions of the second mean field on the oscillators. This would exactly correspond to the particular model we study theoretically below.

**Figure 1:**
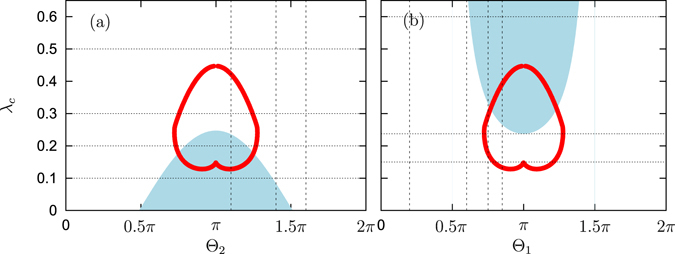
Stability of the incoherent state and the end points of anomalous branches. Blue region: stability of the incoherent state according to formula (16). Red lines: points on plane of parameters $({{\rm{\Theta }}}_{\mathrm{1,2}},\lambda )$, where *R* = 0. These are the points (calculated according to formula (28)) where the “anomalous” branch has vanishing order parameter. Panel (a): case ${{\rm{\Theta }}}_{1}=0$; panel (b): case ${{\rm{\Theta }}}_{2}=0$. Dotted lines show values of *λ* for which the diagrams in Figs 3 and 5 are plotted. Dashed lines show values of ${{\rm{\Theta }}}_{1},{{\rm{\Theta }}}_{2}$ for which the diagrams in Figs 4 and 6 are plotted.

We focus below on the simplest possible setup, where all oscillators that are described in the phase approximation have the same natural frequency; furthermore, the coupling is assumed to involve the first harmonics of the oscillations only, like in the standard Kuramoto-Sakaguchi model. We show that mostly nontrivial dynamics of this system is observed if the two mean fields act differently, i.e. one is mainly attractive while another one is repulsive. We find uniformly rotating (traveling wave) solutions and study their stability in the thermodynamic limit, where also the Ott-Antonsen approach can be applied^26^. In the case traveling waves are unstable, modulated traveling waves are observed. The findings are supported by direct numerical simulations of finite ensembles.

## Results

### Model formulation

In this paper we consider a simple model of a population of phase oscillators subject to a coupling through two mean fields. We start by formulating the problem in a rather general context, but will make several simplifications to achieve a tractable but still nontrivial model. We assume that all the phase oscillators have the same natural frequency, and differ only by the way how they contribute to the mean fields and how they are forced by them. Furthermore, we assume that these differences are only in the phases of the coupling, not in the amplitudes. Physically, this can be modeled by an ensemble of acoustic oscillators, sounds of which are collected by two microphones, and which are subject to the forcing emitted by two loudspeakers. If the positions of the oscillators are different, they contribute to the mean fields with different phase shifts and get signals that are also differently phase shifted. On the other hand, attenuation of signals can be neglected, thus only the phase relations are important.

We denote the phases of the oscillators *ϕ*_*k*_, *k* = 1, …, *N*, and define two complex mean fields *Y* ^(1,2)^ according to

1 $${Y}^{\mathrm{(1,2)}}=\frac{1}{N}\sum _{j=1}^{N}{e}^{i{\varphi }_{j}+i{\gamma }_{j}^{\mathrm{(1,2)}}}$$

where *N* is the number of oscillators in the system, and ${\gamma }_{j}^{\mathrm{(1,2)}}$ are the phase shifts with which the oscillators contribute to the mean fields. The dynamics of the phases, driven by these fields, is given by equations (written in the reference frame rotating with the common frequency)

2 $${\dot{\varphi }}_{k}={\varepsilon }_{1}{\rm{Im}}({Y}^{\mathrm{(1)}}{e}^{-i{\varphi }_{k}-i{\delta }_{k}^{\mathrm{(1)}}})+{\varepsilon }_{2}{\rm{Im}}({Y}^{\mathrm{(2)}}{e}^{-i{\varphi }_{k}-i{\delta }_{k}^{\mathrm{(2)}}})$$

where *ε*_1,2_ are the coupling constants of the two fields, and ${\delta }_{k}^{\mathrm{(1,2)}}$ are the phase shifts with which the fields act on oscillators.

Generally, the model above would be fully defined if the joint distribution density of the phase shifts $W({\gamma }^{\mathrm{(1,2)}},{\delta }^{\mathrm{(1,2)}})$ is given. To simplify, we assume that the parameters ${\gamma }^{\mathrm{(1,2)}},{\delta }^{\mathrm{(1,2)}}$ are independent on each other in the populations. Due to this, as one can see from (2), the dynamics of *ϕ*_*k*_ does not depend on the phase shifts ${\gamma }^{\mathrm{(1,2)}}$ and one can simplify the expressions for the mean fields (1) as

3 $${Y}^{\mathrm{(1,2)}}=\langle {e}^{i\varphi }\rangle \langle {e}^{i{\gamma }^{\mathrm{(1,2)}}}\rangle =Z{w}^{\mathrm{(1,2)}}\exp [i{\mu }^{\mathrm{(1,2)}}]$$

where we introduced the usual Kuramoto complex mean field and two complex constants characterizing the distributions of the phase shifts ${\gamma }^{\mathrm{(1,2)}}$:

4 $$Z=\langle {e}^{i\varphi }\rangle =\frac{1}{N}\sum _{j}{e}^{i{\varphi }_{j}},{w}^{\mathrm{(1,2)}}\exp [i{\mu }^{\mathrm{(1,2)}}]=\langle {e}^{i{\gamma }^{\mathrm{(1,2)}}}\rangle $$

The dynamics of the phases (2) remains depending on the distributions of ${\delta }_{k}^{\mathrm{(1,2)}}$. Below we consider a minimal nontrivial case, when only one set of the phase shifts ${\delta }_{k}^{\mathrm{(1,2)}}$ has a nontrivial distribution (say, the second one), while another set consists of equal shifts ${\delta }^{\mathrm{(1)}}$. Then we can rewrite Eq. (2) as

5 $${\dot{\varphi }}_{k}={\varepsilon }_{1}{w}^{\mathrm{(1)}}{\rm{Im}}(Z{e}^{-i{\varphi }_{k}-i{\delta }^{\mathrm{(1)}}+i{\mu }^{\mathrm{(1)}}})+{\varepsilon }_{2}{w}^{\mathrm{(2)}}{\rm{Im}}(Z{e}^{-i{\varphi }_{k}-i{\delta }_{k}^{\mathrm{(2)}}+i{\mu }^{\mathrm{(2)}}})$$

We renormalize time $t{\varepsilon }_{2}{w}^{\mathrm{(2)}}\to t$ and obtain finally the basic model that we will study in this paper:

6 $${\dot{\varphi }}_{k}=\lambda {\rm{Im}}(Z{e}^{-i{\varphi }_{k}+i{\Theta }_{1}})+{\rm{Im}}(Z{e}^{-i{\varphi }_{k}+i{\alpha }_{k}+i{{\rm{\Theta }}}_{2}}),Z=R{e}^{i{\rm{\Psi }}}=\langle {e}^{i\varphi }\rangle $$

where we introduced a real parameter $\lambda =\frac{{\varepsilon }_{1}{w}^{\mathrm{(1)}}}{{\varepsilon }_{2}{w}^{\mathrm{(2)}}}$ describing the relative strengths of couplings of the two mean fields; ${{\rm{\Theta }}}_{1}={\mu }^{\mathrm{(1)}}-{\delta }^{\mathrm{(1)}}$ is the effective constant phase shift for the first mean field; ${{\rm{\Theta }}}_{2}$ is the phase at which the distribution of the phase shift of the second field ${\mu }^{\mathrm{(2)}}-{\delta }_{k}^{\mathrm{(2)}}$ has a maximum; and finally the parameter ${\alpha }_{k}={\mu }^{\mathrm{(2)}}-{\delta }_{k}^{\mathrm{(2)}}-{{\rm{\Theta }}}_{2}$ describes the deviation of the phase shift from this most probable one. Below we use the von Mises distribution of phase shifts *α*

7 $$g(\alpha )=\frac{\exp [{\rm{\Delta }}\,\cos \,\alpha ]}{2\pi {I}_{0}({\rm{\Delta }})}$$

characterized by the parameter Δ: Δ = 0 corresponds to a uniform distribution of the phase shifts, while in the limit Δ → ∞ one gets a delta-function.

It is instructive to rewrite Eq. (6) in the real form:

8 $${\dot{\varphi }}_{k}=\lambda R\,\sin ({\rm{\Psi }}-{\varphi }_{k}+{{\rm{\Theta }}}_{1})+R\,\sin ({\rm{\Psi }}-{\varphi }_{k}+{{\rm{\Theta }}}_{2}+{\alpha }_{k}),R{e}^{i{\rm{\Psi }}}=\langle {e}^{i\varphi }\rangle $$

The physical interpretation of this two-mean-field-coupling model (8) is as follows: each of the couplings is of Sakaguchi-Kuramoto type, i.e. it contains the sin term only. The two couplings have different phase shifts, for the first coupling it is fixed to ${{\rm{\Theta }}}_{1}$, for the second coupling the phase shift is different for different oscillators, but is concentrated around ${{\rm{\Theta }}}_{2}$. Parameter *λ* defines the relative weight of the two couplings. The main parameters of the problem are $\lambda ,{{\rm{\Theta }}}_{1},{{\rm{\Theta }}}_{2}$, and Δ. One can expect mostly nontrivial effects if the two couplings act in opposite directions: one tries to synchronize the oscillators, while another one is repulsive and tends to desynchronize them. Below we will keep all the parameters in the theoretical considerations, but in numerical examples we will mainly set either ${{\rm{\Theta }}}_{1}=0$, that corresponds to a purely attractive first coupling, or ${{\rm{\Theta }}}_{2}=0$, that corresponds to a purely attractive second coupling.

### Stability of incoherent state

First, we consider stability properties of the completely incoherent state where the phases of all the oscillators are uniformly distributed and the mean field vanishes. Analytical expression for the growth rate of potentially unstable perturbations, derived in the section Methods, reads

9 $${\rm{Re}}(\gamma )=\frac{\lambda {\cos {\rm{\Theta }}}_{1}}{4}+\frac{{I}_{1}({\rm{\Delta }}){\cos {\rm{\Theta }}}_{2}}{4{I}_{0}({\rm{\Delta }})}$$

It determines critical coupling parameter *λ*_c_ at which the incoherent state becomes unstable (Re $(\gamma ) > 0$). For example, for ${{\rm{\Theta }}}_{1}=0$ (attractive first coupling) we have ${\lambda }_{c}=-\,{\cos {\rm{\Theta }}}_{2}\frac{{I}_{1}({\rm{\Delta }})}{{I}_{0}({\rm{\Delta }})}$, this boundary is depicted in Fig. 1(a). One can see that stability of the incoherent state is only possible if the second coupling is repulsive (i.e. ${{\rm{\Theta }}}_{2}$ is close to *π*) and relatively strong (*λ* is small). In panel (b) we show the case when ${{\rm{\Theta }}}_{2}=0$ is fixed, here the stability boundary of the incoherent state is given by ${\lambda }_{c}=-\,\frac{{I}_{1}({\rm{\Delta }})}{{I}_{0}({\rm{\Delta }}){\cos {\rm{\Theta }}}_{1}}$. In this figure there is another nontrivial line to be discussed below in the next section.

### Traveling wave solutions and their stability

Here we discuss nontrivial regimes of partial synchrony in the model. We use the self-consistent approach to find the solutions; reformulate the system in terms of Ott-Antonsen (OA) equations; and then determine stability of the solutions by calculating their stability spectra. The details of these methods are described in the Methods Section, here we summarize the results.

The traveling wave synchronous solution have the mean field with a constant amplitude *R* and a uniformly rotating phase ${\rm{\Psi }}={\rm{\Omega }}t+{{\rm{\Psi }}}_{0}$. The distribution of the phases is stationary in the reference frame rotating with Ω. Driven by the two mean fields, some oscillators are locked (i.e. they rotate with the same frequency Ω), while others are not locked and rotate (although non-uniformly) with some other frequencies. The main parameters we use to characterize the state of the system, are the amplitude *R* and the locking parameter *P*_*l*_ (cf. Eq. (25)), determining which portion of oscillators in the population is locked (*P*_*l*_ = 1 means all are locked; *P*_*l*_ = 0 means no one is locked). For determination of stability, we use a reformulation of the dynamics in terms of the Ott-Antonsen integro-differential system of equations (see description in Methods Section, in particular Eq. (26)); the latter equation is linearized and discretized to find the spectrum of eigenvalues.

**Figure 2:**
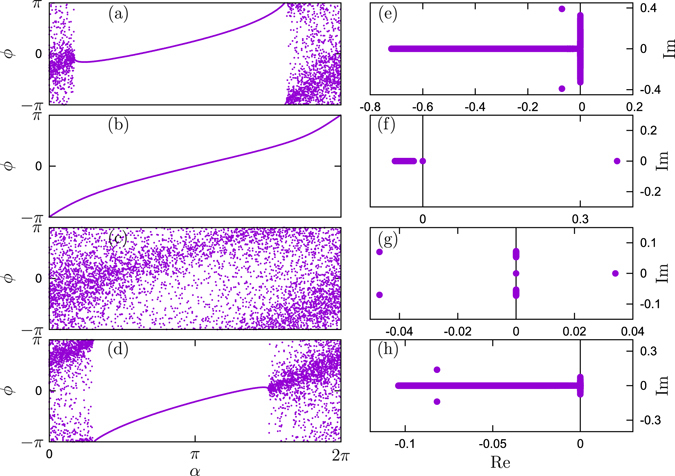
Synchronous states and their stability. Phase profiles (left panels) and their stability spectra (right panels) for the constructed synchronization states. Panels (a,b,e,f) show the case of attractive first coupling. The parameters here are ${{\rm{\Theta }}}_{1}=0$, ${{\rm{\Theta }}}_{2}=-\,0.9\pi$, and $\lambda =0.45$, with (**a**): $R=0.295$ and the locking parameter ${P}_{l}=0.61$ (normal branch); and (**b**): $R=0.02$ and ${P}_{l}=1$ (anomalous branch). (e,f): correspondent stability spectra of these states. Vertical line shows the imaginary axis, for better identification of instability. Panels (c,d,g,h) show the case of attractive first coupling with parameters ${{\rm{\Theta }}}_{1}=0$, ${{\rm{\Theta }}}_{2}=-\,0.9\pi$ and $\lambda =0.2$, with (**c**): $R=0.028$ and ${P}_{l}=0$ (normal branch); and (**d**) $R=0.07$ and ${P}_{l}=0.45$ (anomalous branch).

In Fig. 2 we present some typical stationary synchronous states together with their stability spectra. In these figures we visualize the stationary density via a dispersion of a finite set of points, for better visibility. One can easily distinguish domains of locked (all points representing oscillators collapse to a line) and rotating (scattered) oscillators. We must emphasize that the partial phase locked state here is not a chimera state, as the oscillators react to the fields with different phase shifts, and therefore there is no structural symmetry in the system which has to be broken in the chimera phenomenon. Panels (a), (c), (e) and (g) show the profile of normal branches which can also be found in one mean field coupling cases, while the anomalous branches shown in panel (b), (d), (f) and (h), do not appear in the traditional one mean field case. One remarkable thing is that, the ratio of phase locked oscillators in (b) is larger than in (a), but the order parameter in (b) is smaller than in (a). This is also a specific effect of the two mean field coupling, compared to the usual one mean field coupling.

One can see from Fig. 2 that the spectrum generally consists of a continuous part and several discrete eigenvalues. The continuous part is related to existence of the branch of the locked oscillators (a set of purely real eigenvalues) and to the unlocked oscillators (a set of purely imaginary eigenvalues). In the case both branches are present (panels (a,d) of Fig. 2), one has a characteristic T-shaped continuous (essential) spectrum, as argued in refs 27 and 28 for the chimera states, where also locked and unlocked oscillators are present. The state in panel (b) of Fig. 2 is fully locked, here only the real continuous spectrum is present. In the case (c) of Fig. 2, where all the oscillators are unlocked, the purely imaginary continuous spectrum is observed.

As argued in refs 27 and 28 stability is determined by the discrete spectrum, which is clearly seen for all cases: panels (a,d) depict stable solutions, while panels (b,c) depict unstable ones. In some cases (not illustrated here) it is difficult to distinguish the discrete spectrum from the continuous one, as the discrete eigenvalues have very small real part and are “smeared” (i.e. depend significantly on the offset parameter *α*_0_). Here refining the resolution (i.e. increasing the number of discretization points *L*) helps, but the computation time increases rapidly.

### Normal, anomalous, and hybrid states

Basing on the approach described above, we have determined the uniformly rotating states for different values of parameters, and characterized their stability. Here below we describe the main types of the solutions.

#### Attractive first coupling

**Figure 3:**
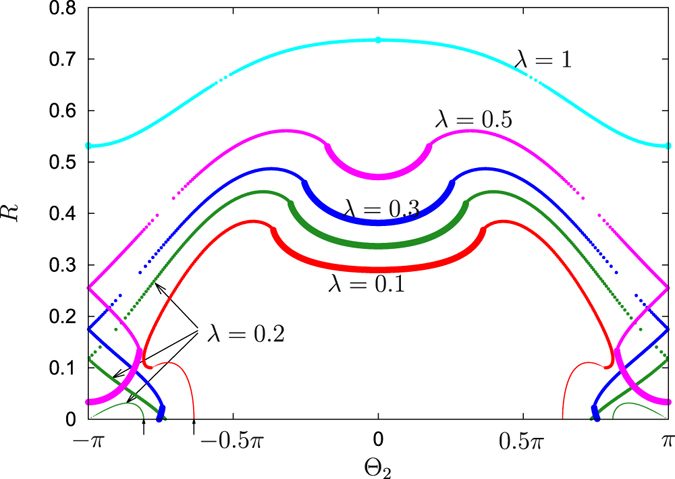
Synchronous states for attractive first coupling. Order parameter *R* for ${{\rm{\Theta }}}_{1}=0$ and ${\rm{\Delta }}=0.5$, as functions of ${{\rm{\Theta }}}_{2}$ for several values of *λ*. Large markers: states with all *P*_*l*_ = 1 (all oscillators locked); medium markers: states that are partially locked $0 < {P}_{l} < 1$; small markers: all oscillators are unlocked *P*_*l*_ = 0. Arrows at the *x*-axis show the linear stability boundaries for *λ* = 0.1 and for *λ* = 0.2, according to Eq. (16).

**Figure 4:**
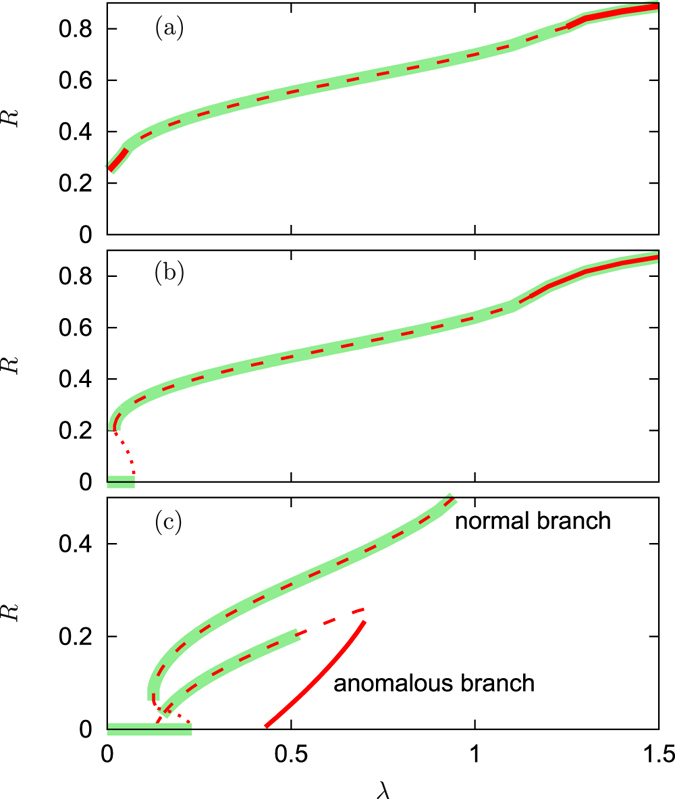
Normal and anomalous branches for attractive first coupling. The global order parameter of traveling wave states as functions of *λ*. The parameters are ${{\rm{\Theta }}}_{1}=0$, ${\rm{\Delta }}=0.5$, and the values of ${{\rm{\Theta }}}_{2}$ are (**a**): ${{\rm{\Theta }}}_{2}=-\,0.4\pi$; (**b**): ${{\rm{\Theta }}}_{2}=-\,0.6\pi$; (**c**): ${{\rm{\Theta }}}_{2}=-\,0.9\pi$. Bold lines: states with all *P*_*l*_ = 1 (all oscillators locked); dashed lines: states that are partially locked $0 < {P}_{l} < 1$; dotted lines: all oscillators are unlocked *P*_*l*_ = 0. Stable traveling wave and incoherent solutions are highlighted with bold green line in background.

In this subsection we present the results for the case ${{\rm{\Theta }}}_{1}=0$, i.e. the first (non-distributed) coupling is purely attractive. We present the dependencies of the order parameter *R* on ${{\rm{\Theta }}}_{2}$, obtained using the self-consistent method above, in Fig. 3. To fix ${{\rm{\Theta }}}_{1}=0$, in relations (21) we varied parameter *β* in the range $0\le \beta  < 2\pi$ and found all the points with $Q={{\rm{\Theta }}}_{1}=0$. These points yield a parametric representation of the order parameter *R* and of ${{\rm{\Theta }}}_{2}$ as functions of the remaining auxiliary parameter *a*. Additionally, we calculated the portion of locked oscillators according to (25) and coded it in Fig. 3 with the size of the markers into three types: all oscillators locked (*P*_*l*_ = 1), all oscillators unlocked (*P*_*l*_ = 0), and partial locking ($0 < {P}_{l} < 1$). First, one can notice the symmetry of the diagram Fig. 3 ${{\rm{\Theta }}}_{2}\to -\,{{\rm{\Theta }}}_{2}$, this is the consequence of the selected value ${{\rm{\Theta }}}_{1}=0$. Therefore, for the analysis of the dependence of the order parameter *R* on *λ*, presented in Fig. 4, we choose only negative values of the phase shift ${{\rm{\Theta }}}_{2}$. In Fig. 4 we also use the style of the lines (solid, dashed, dotted) to distinguish fully locked, partially locked, and fully unlocked states.

The situation is quite simple for ${{\rm{\Theta }}}_{2}\approx 0$: here both mean fields act attractively and the dependence on the parameter *λ* is monotonous (see the curve for ${{\rm{\Theta }}}_{2}=-\,0.4\pi$ in Fig. 4(a)). Remarkably, here solutions with small value of *λ*, where the second mean field dominates, are fully locked, while at medium values of *λ*, where both fields compete, only partial locking is observed. At large *λ* the attractive first coupling dominates again, and here *P*_*l*_ = 1.

For phase shifts ${{\rm{\Theta }}}_{2}$ that are closer to $-\pi$, instead of a monotonous dependence of the order parameter *R* on *λ*, we obtain a dependence characteristic for a subcritical transition of the first-order type, see the curve for ${{\rm{\Theta }}}_{2}=-\,0.6\pi$ in Fig. 4(b). Stability consideration reveals that in the region where two nontrivial solutions exist, solutions with lower value of *R* are unstable and those with higher value of *R* are stable. Thus, we have a typical diagram for a hysteresic transition of the first order: there is a range of parameter *λ* where the incoherent and coherent solutions are both stable, while “in between” there is an unstable solution. When this unstable solution meets the incoherent one (see arrows below the ${{\rm{\Theta }}}_{2}$-axis in Fig. 3) the latter loses its stability. It is worth mentioning that the large part of the unstable coherent solution is fully unlocked, although a part of it can be partially locked (e.g., this happens for ${{\rm{\Theta }}}_{2}=-\,0.9\pi$, see Fig. 4(c)).

The situation for ${{\rm{\Theta }}}_{2}$ even closer to −*π*, illustrated by the results for Θ_2_ = −0.9*π* in Fig. 4(c), is rather unusual and deserves a more detailed description. First, one can see here the branch which is very much similar to the solution for Θ_2_ = −0.6*π*, only it is shifted to larger values of parameter *λ*. Here the stability properties are similar to that at Θ_2_ = −0.6*π*: solutions with $dR/d\lambda  > 0$ are stable while the state with $dR/d\lambda  < 0$ is unstable. The latter unstable part of this branch appears exactly at the linear stability border point. We call this branch “normal branch”, as it is very similar to the situation observed at a usual subcritical transition.

One can see that additionally there exists another branch of solutions with relatively small values of *R*. Noteworthy, the values of *λ* at which this branch emerges from *R* = 0, have nothing to do with the change of linear stability of the incoherent state. Therefore we call this branch “anomalous”. The anomalous solutions can also be seen in Fig. 3, they do not end at the points (marked by arrows) where linear stability of the incoherent state changes.

To clarify the behavior of the solutions in the limit R → 0, we need to take this limit for the solutions constructed by Self-consistent approach presented in the Methods Section. There are two possibilities to have vanishing order parameter *R*: one is related to vanishing of *r* in the equation for the phases (19), and another is related to the case Ω = 0. The former, “normal” case corresponds to a uniform distribution of the phases for all *α* in the limit *R* → 0, i.e. to the incoherent state. The values of parameters at which this happens are exactly those at which the linear stability of the incoherent state changes. Thus the “normal” branch bifurcates from the non-coherent state.

In contradistinction, the states with vanishing *R* that correspond to Ω = 0, are not close to the incoherent state with a uniform distribution of phases. Moreover, as Fig. 4(c) shows, one part of the “anomalous” branch consists of states with *P*_*l*_ = 1, i.e. all the oscillators are locked. Therefore we cannot describe the appearance of the “anomalous” branch as a bifurcation from the incoherent solution.

Stability analysis of the anomalous branch shows that the solutions with *P*_*l*_ = 1 are unstable, while another part with $0 < {P}_{l} < 1$ consists of unstable and stable solutions. The change of stability happens without appearance of new traveling wave solutions, although we cannot exclude that some other solutions (e.g., modulated traveling waves) may appear at these points.

#### Attractive second coupling

**Figure 5:**
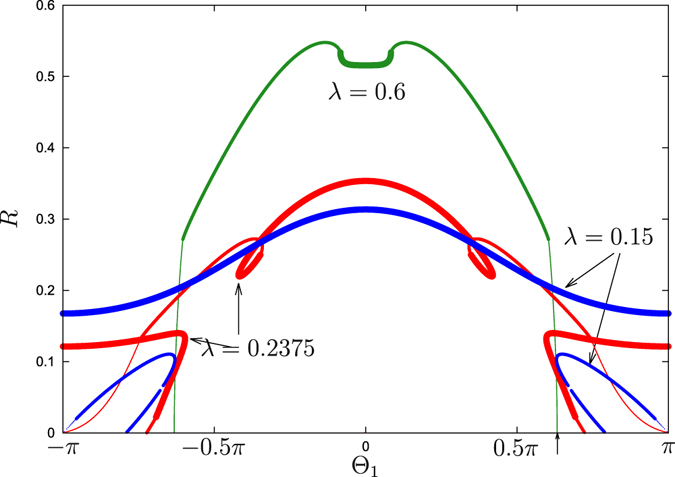
Synchronous states for attractive second coupling. The order parameter *R* for ${{\rm{\Theta }}}_{2}=0$ and ${\rm{\Delta }}=0.5$, as functions of ${{\rm{\Theta }}}_{1}$ for several values of *λ*. Large markers: states with all *P*_*l*_ = 1 (all oscillators locked); medium markers: states that are partially locked 0 < *P*_*l*_ < 1; small markers: all oscillators are unlocked *P*_*l*_ = 0. Arrows at the *x*-axis show the linear stability boundaries for *λ* = 0.6 according to Eq. (16).

**Figure 6:**
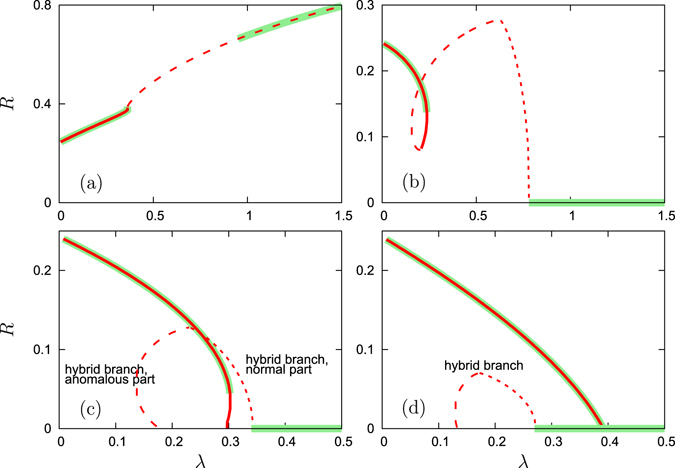
Normal, anomalous, and hybrid branches for attractive second coupling. The global order parameter of traveling wave states as functions of *λ*. The parameters are ${{\rm{\Theta }}}_{2}=0$ and ${\rm{\Delta }}=0.5$, and the values of ${{\rm{\Theta }}}_{1}$ are (**a**): ${{\rm{\Theta }}}_{1}=0.2\pi$; (**b**): ${{\rm{\Theta }}}_{1}=0.6\pi$; (**c**): ${{\rm{\Theta }}}_{1}=0.75\pi$; (**d**): ${{\rm{\Theta }}}_{1}=0.85\pi$. Bold lines: states with all ${P}_{l}=1$ (all oscillators locked); dashed lines: states that are partially locked $0 < {P}_{l} < 1$; dotted lines: all oscillators are unlocked ${P}_{l}=0$. Stable traveling wave and incoherent solutions are highlighted with bold green line in background.

Here we discuss a situation where the phase shift of the second coupling is fixed ${{\rm{\Theta }}}_{2}=0$, while the phase shift of the first coupling ${{\rm{\Theta }}}_{1}$ varies. Figures 5 and 6 are similar to the Figs 3 and 4 in the previous subsection. Figure 5 shows nontrivial states when the first and the second couplings act in different directions, i.e. when ${{\rm{\Theta }}}_{1}$ is close to *π*. Let us focus on the dependence of the order parameter *R* on the coupling constant *λ* in Fig. 6. For small values of ${{\rm{\Theta }}}_{1}$, the incoherent state is unstable for all *λ*, and a traveling wave solution exists - however it is unstable for intermediate values of *λ*, see Fig. 6(a). For larger values of ${{\rm{\Theta }}}_{1} > \pi \mathrm{/2}$, where the first coupling is repulsive, the incoherent state becomes stable at large *λ* (see Fig. 6(b), here ${{\rm{\Theta }}}_{1}=0.6\pi$). When *λ* decreases, at the instability border the normal branch of solutions softly appears with small values of *R*. With decreasing of *λ* this branch makes a loop, and only for small *λ* a part of this branch becomes stable. For larger values of ${{\rm{\Theta }}}_{1}$, the loop becomes larger and eventually crosses the axis *R* = 0 at two points (which correspond to the situations where anomalous solutions have vanishing order parameter, these points are marked red in Fig. 1(b)); see also panels (c,d) in Fig. 6. Remarkably, as a result the branch which starts as a normal branch, now ends as an anomalous one, thus it can be termed “hybrid branch”. Another anomalous branch that consists of solutions with *P*_*l*_ = 1, ends at *λ* = 0. The anomalous and the hybrid branches exist also for ${{\rm{\Theta }}}_{1}=0.85\pi$, see Fig. 6(d).

Noteworthy, panels (a,b,c) of Fig. 6 show stability gap: there are intermediate values of the coupling constant *λ* at which there are no stable traveling wave solutions. Here numerical simulations, presented in the next sections, demonstrate time-periodic regimes (modulated traveling waves). In Fig. 6(d) there is a bistable region where both the anomalous and the incoherent solutions are stable, here a hysteresic transition is observed.

### Simulation of the Ott-Antonsen system

**Figure 7:**
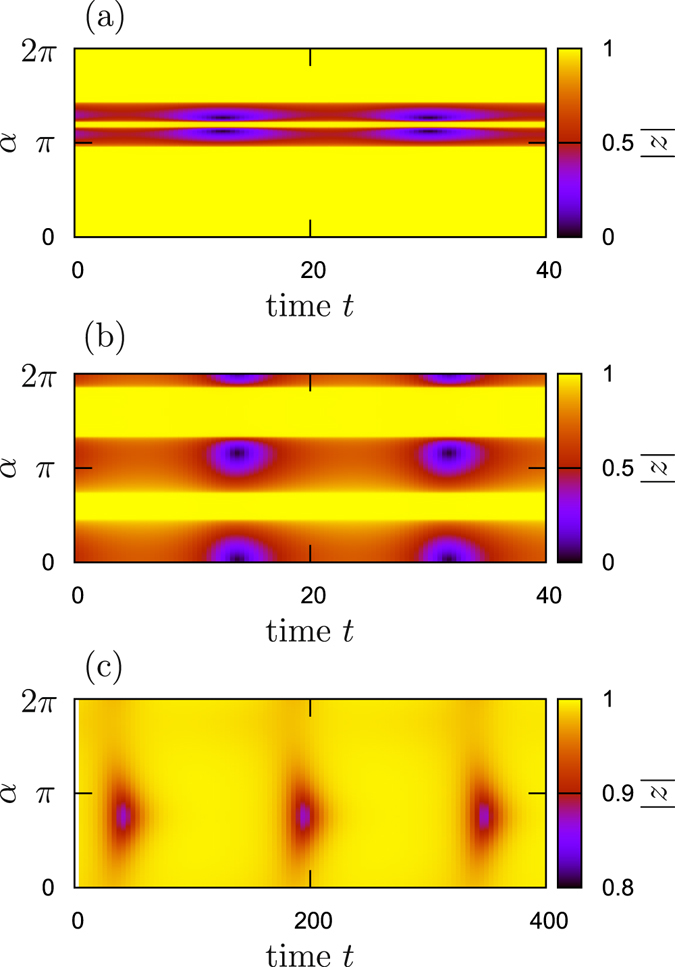
Modulated solutions in simulations of Ott-Antonsen equations. Periodic in time evolution of the local order parameter $|z(\alpha ,t)|$ for situations where stationary traveling waves are unstable. Panel (a): ${{\rm{\Theta }}}_{1}=0.2\pi$, ${{\rm{\Theta }}}_{2}=0$ and $\lambda =0.7$. Panel (b): ${{\rm{\Theta }}}_{1}=0.6\pi$, ${{\rm{\Theta }}}_{2}=0$ and $\lambda =0.5$. Panel (c): ${{\rm{\Theta }}}_{1}=0.75\pi$, ${{\rm{\Theta }}}_{2}=0$ and $\lambda =0.315$. Simulations started from random initial conditions, the transient stage is omitted. The same parameter values are used in Fig. 10.

In this and the next section we report on the numerical tests of the found solutions. The first test deals with the system in the thermodynamic limit. Here, according to the Ott-Antonsen ansatz^26^, one can write for each *α* a closed equation for the local order parameter $\langle {e}^{i{\psi }_{\alpha }}\rangle$ and thus to represent the whole system as an integro-differential equation (eq. (26) in the Method Section). The simulation is accomplished via discretization of the integral and by solving the resulting finite-dimensional system with the Runge-Kutta method. In the cases, when at least one stable traveling wave solution is present, direct numerical simulations confirm the stability results above. Thus we present only the nontrivial cases where no stable traveling wave solution exists (see Fig. 6 above). In Fig. 7 we show the patterns appearing in these situations. The dynamics of the order parameter $|z(\alpha ,t)|$ is periodic in time. Together with rotation of the phase this gives quasiperiodic dynamics (see the snapshots of the modulated synchronous states below where the same patterns are checked in finite-size ensemble simulations), i.e. a modulated traveling wave. Remarkably, patterns in panels (a,b) of Fig. 7 show two locked and two unlocked regions. Modulated wave in panel (c) of Fig. 7 can be characterized as a “blinking locked state”, as here for large time intervals the local order parameter is close to one everywhere, and only for relatively short time intervals, weakly unlocked regions, where this order parameter is reduced to $|z|\approx 0.9$, are observed.

### Direct numerical simulations of oscillator population

In this section, we present results for numerical simulations of a finite size oscillator system, Eq. (8). First, we present results for a large system: N = 10000. Figure 2 in the Supplementary Information shows both the results from the self-consistent solution and direct simulation result with the parameter ${{\rm{\Theta }}}_{1}=0$ (attractive first mean field). The results confirm our solutions in the thermodynamic limit, presented in Fig. 3.

**Figure 8:**
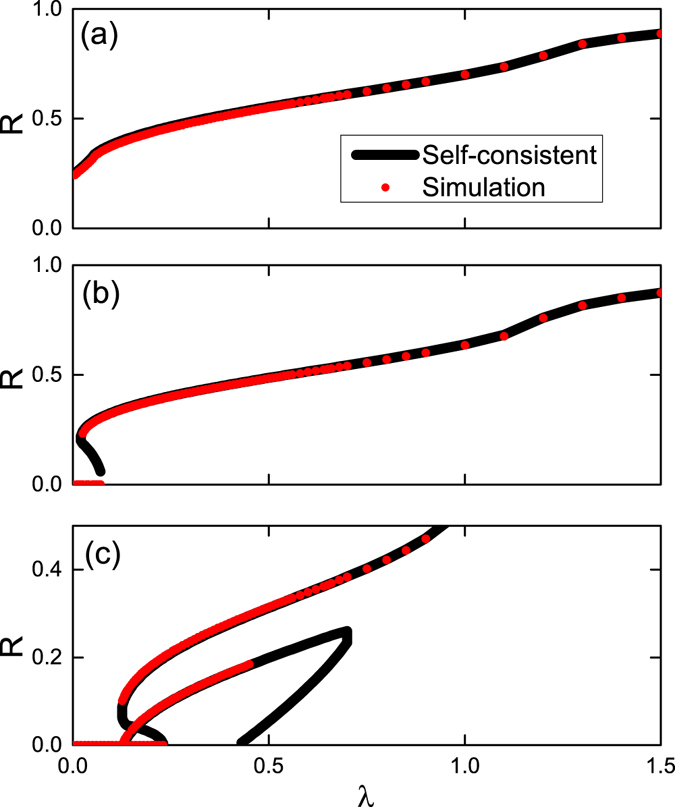
Direct numerical simulations for attractive first coupling. The global order parameter of traveling wave states as functions of *λ*. The parameters are ${{\rm{\Theta }}}_{1}=0$, ${\rm{\Delta }}=0.5$, and the values of ${{\rm{\Theta }}}_{2}$ are (**a**): Θ_2_ = −0.4*π*; (**b**): Θ_2_ = −0.6*π*; (**c**): Θ_2_ = −0.9*π*. Black lines represent the self-consistent solution and red circles represent the direct simulation results.

Figure 8 compares the self-consistent solutions and the simulation results for the traveling wave states shown in 4 above. The parameters are Θ_1_ = 0, ${\rm{\Delta }}=0.5$ and Θ_2_ = (a): −0.4*π*; (b): −0.6*π*; (c): −0.9*π*. The simulation results show the normal and the anomalous solutions in Fig. 4, and also confirm our previous results.

**Figure 9:**
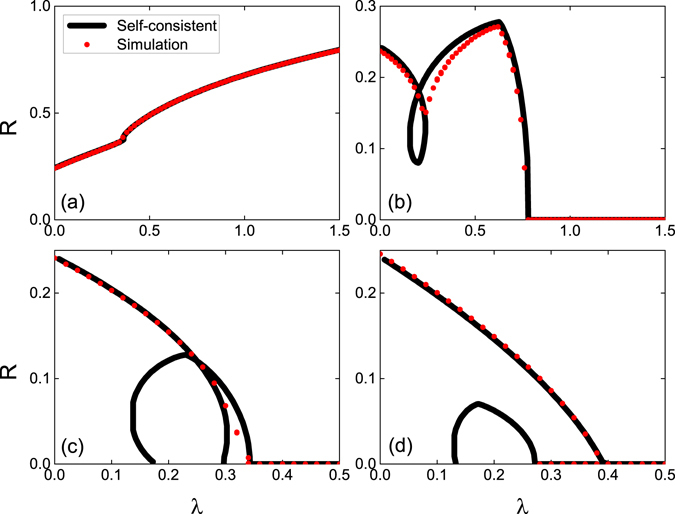
Direct numerical simulations for attractive second coupling. The global order parameter of traveling wave states as functions of *λ*. The parameters are ${{\rm{\Theta }}}_{2}=0$, ${\rm{\Delta }}=0.5$, and the values of ${{\rm{\Theta }}}_{1}$ are (**a**): ${{\rm{\Theta }}}_{1}=0.2\pi$; (**b**): ${{\rm{\Theta }}}_{1}=0.6\pi$; (**c**): ${{\rm{\Theta }}}_{1}=0.75\pi$; (**d**): ${{\rm{\Theta }}}_{1}=0.85\pi$. Black lines represent the self-consistent solution and red circles represent the direct simulation results.

**Figure 10:**
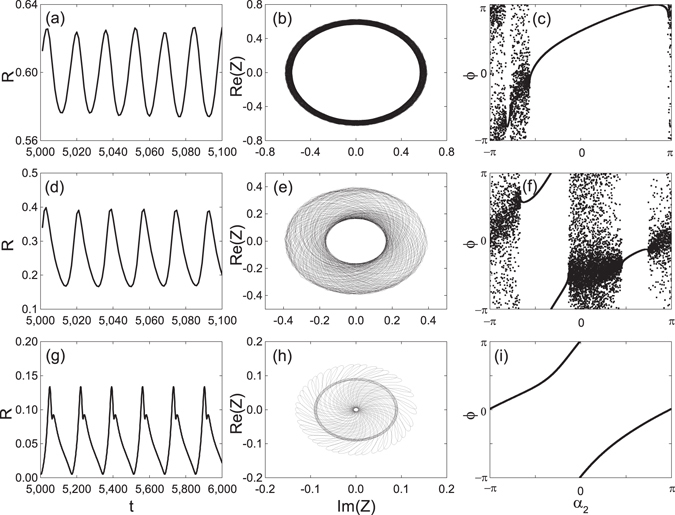
Modulated synchronous states in direct numerical simulations. Simulations for the parameters where no stable traveling waves exist, with ${{\rm{\Theta }}}_{2}=0$ and ${\rm{\Delta }}=0.5$. Panels (a), (d) and (g) show the time dependence of the global order parameter *R*, after a long transient is discarded. Panels (b), (e) and (h) show the real and the imaginary part of the complex global order parameter *Z*. Panels (c), (f) and (i) show the snapshot of the phases of oscillators. The parameters are: in (**a**), (**b**) and (**c**), ${{\rm{\Theta }}}_{1}=0.2\pi$, $\lambda =0.7$; in (**d**), (**e**) and (**f**), ${{\rm{\Theta }}}_{1}=0.6\pi$, $\lambda =0.5$; in (**g**), (**h**) and (**i**), ${{\rm{\Theta }}}_{1}=0.75\pi$, $\lambda =0.315$.

Figure 9 shows the simulation results for traveling wave states depicted in Fig. 6 above. The parameters are ${{\rm{\Theta }}}_{2}=0$, ${\rm{\Delta }}=0.5$ and ${{\rm{\Theta }}}_{1}$= (a): $0.2\pi$; (b): $0.6\pi$; (c): $0.75\pi$; (d): $0.85\pi$. The simulation results shows the normal, the anomalous, and the hybrid branches in Fig. 6, and also confirm our stability analysis. In the cases where no stable traveling waves exist, time-averaged values of the order parameter are shown. To reveal the modulated solutions, we show in Fig. 10 the details of the system behavior for cases presented in Fig. 7. Panels (a), (d) and (g) show periodic in time evolution of the global order parameter *R*. Panels (b), (e) and (h) demonstrate quasiperiodic rotations of the complex order parameter, confirming that the observed regimes are modulated traveling waves. In panel (c), (f) and (i), one can see that the oscillators are partial locked. In fact, in panel (i) one cannot recognize the unlocked pattern, because at the moment of time when the snapshot has been performed, the local order parameter is everywhere close to one. Only during small time epochs over the period the unlocked region is visible (see discussion of “blinking unlocking” above.)

Next, we study systems with a small number oscillators. Here we focus mainly on situations of multistability, to check whether all the states stable in the thermodynamic limit can be also observed for small populations. Figure 3 in the Supplementary Information shows the solutions for *N* = 100 and the same parameters as in Figs 4 and 8 above (parameters are ${{\rm{\Theta }}}_{1}=0$, ${\rm{\Delta }}=0.5$, and Θ_1_ = (a): −0.4*π*; (b): −0.6*π*; (c): −0.9*π*). For each *λ*, we plot the final order parameters generated from 1500 random initial conditions. Comparing with the results for a large ensemble *N* = 10000 (Fig. 8), one can see that for the cases of ${{\rm{\Theta }}}_{2}=-\,0.4\pi$ and ${{\rm{\Theta }}}_{2}=-\,0.6\pi$, a small system shows the same behavior as a large one. However, for the case of ${{\rm{\Theta }}}_{2}=-\,0.9\pi$, we miss the anomalous solution in the small system, only the normal branch is observed. We conclude that the basin of this anomalous solution is rather small, so possibly finite-size fluctuations in a small system lead to a transition to the normal branch.

Figure 4 in the Supplementary Information shows the probability of a finite-time system to end up in the incoherent state, calculated for 50 realizations with randomly chosen initial conditions. Parameters are ${{\rm{\Theta }}}_{1}=0$, ${\rm{\Delta }}=0.5$ and $\lambda =0.2$ (see the green curve in Fig. 3 for the solutions in the thermodynamic limit). The system sizes are (a): *N* = 10000; (b): *N* = 5000, (c): *N* = 1000, and (d): *N* = 500; the red lines show the linear stability boundaries according to Eq. (16). One can see that with the increasing of system size, the probability of getting incoherent state are closer to 1 and the stability boundaries are also closer to the theoretical results. For system size ensembles, the probability of getting incoherent state becomes lower. This can be explained by the fluctuations of the order parameter, which increase with the decreasing system size, so the system can jump out of the basin of the incoherent state due to the fluctuations. The nontrivial traveling wave state is relatively stable for these parameters and survives finite-size fluctuations.

## Discussion

In this paper, we studied the dynamics of a population of oscillators driven by two mean fields via two different channels. We have focused on the simplest setup, where all the oscillators have identical frequencies, and the actions of the two mean fields differ by the coupling strengths and by the distributions of coupling phase shifts. The most close situation, previously discussed in the literature, is that of the standard globally coupled ensemble of oscillators (e.g., in the Kuramoto-Sakaguchi formulation) with an additional external feedback^20–24^. In these studies, however, it has been assumed that both channels act with constant, non-distributed coupling phases, what allowed for an essential reduction to a single-channel coupling. Another situation close to the considered in this paper, is that of star-coupled oscillators (i.e. all oscillators are coupled to one central, which then represents one channel of global coupling) with an additional global mean field^29^.

We have presented a general theory of traveling wave solutions in the thermodynamic limit, including study of the stability in the Ott-Antonsen approximation. Mostly nontrivial states are those where the two mean fields act oppositely, i.e. one tends to synchronize the oscillators and another desynchronizes them. Here we have found different types of solutions: states where all oscillators are locked, states where all oscillators are unlocked, and combined states where a part of oscillators are locked by driving fields, and another part is unlocked (we stress that these states cannot be called chimeras, as here there is no symmetry in the population, because the fields act on different oscillators with different phase shifts).

Stability of the found states has been studied using the Ott-Antonsen approach. The problem can be reduced to a linear integral equation, which after discretization can be formulated as a matrix eigenvalue problem. We have demonstrated existence of the essential continuous spectrum, two parts of which lie on the imaginary axis (for unlocked oscillators) and on the real axis in the stable domain (for locked oscillators). Stability is determined by the discrete spectrum which in most cases can be found rather reliably.

By comparing amplitude behavior and stability of the found traveling wave solutions with the stability properties of the incoherent state, we identified normal and anomalous branches of nontrivial solutions. Normal branches bifurcate from the incoherent state when the latter becomes unstable, typically in a subcritical way. The corresponding transitions are hysteretic and discontinuous, which is similar to recent findings of the discontinuous transition in other synchronization setups^30–35^. Additionally, we have found that some branches of solutions appear with vanishing order parameter at points where no change of stability of the incoherent state occurs. These anomalous branches thus do not bifurcate from the incoherent state. Some parts of the anomalous branches may be stable, and they are observed in the simulations of the Ott-Antonsen equations describing the thermodynamic limit. However, in simulations of small finite ensembles the population “prefers” more robust normal stable branches. Furthermore, we have found hybrid branches, which combine properties of the normal and anomalous ones: such a branch starts from a vanishing order parameter at a point where the incoherent state becomes unstable, but ends at a point where stability of the incoherent state does not change.

Additionally, we have found situations where no stable traveling waves exist. Here numerical simulations, both in the thermodynamic limit and in finite ensembles, demonstrated modulated traveling waves with periodically in time pulsating order parameter. Remarkably, in some cases such a wave looks like blinking unlocking: there are epochs when all oscillators look like being locked, while in other epochs one can clearly see a wide distribution of the phases.

Actually, in the situation which the oscillators are coupled by three or more mean fields, and all the channels act with constant. The system can be reduce to our framework by summing the attractive/repulsive couplings into resultant attractive/repulsive mean field, and then it can be studied base on our results. But if the coupling channels have more complicated forms, and can not be summed into resultant mean fields, then one needs to develop a specific approach for the situation.

In conclusion, the competition between two coupling mean fields can make the system behavior much more complicated than the case with only one channel of global coupling. Further study in this direction would help to understand nontrivial synchronization regimes in complex systems in nature, such as coupled cellular systems and neuronal systems.

## Methods

### Linear stability of the incoherent state

In this section we perform a linear stability analysis of the fully incoherent (i.e. with a uniform distribution of phases) state of system (8) in the thermodynamic limit of infinite number of oscillators. In this limit one starts with a continuity equation for the density function *ρ*(*θ, t*, *α*)

10 $$\frac{\partial \rho }{\partial t}+\frac{\partial (\rho \upsilon )}{\partial \varphi }=0,$$

with the velocity $\upsilon =R\lambda \,\sin ({\rm{\Psi }}-\varphi +{{\rm{\Theta }}}_{1})+R\,\sin ({\rm{\Psi }}-\varphi +{{\rm{\Theta }}}_{2}+\alpha )$ and the mean field

$$R{e}^{i{\rm{\Psi }}}=\int {\int }_{0}^{2\pi }d\varphi \,d\alpha \,{e}^{i\varphi }\rho (\varphi ,t,\alpha )g(\alpha ).$$

Suppose there is a small perturbation from the incoherent state $\rho ={\mathrm{(2}\pi )}^{-1}$, i.e.,

11 $$\rho (\varphi ,t,\alpha )=\frac{1}{2\pi }+\varepsilon \eta (\varphi ,t,\alpha )$$

with $\varepsilon \ll 1$. Expanding the perturbation *η* in the Fourier series

12 $$\eta (\varphi ,t,\alpha )=c(\alpha ,t){e}^{i\varphi }+{c}^{\ast }(\alpha ,t){e}^{-i\varphi }+{c}^{\perp }(\alpha ,t),$$

where ${c}^{\perp }(\alpha ,t)$ is the sum of higher harmonics, we can represent the mean field as

$$R{e}^{i{\rm{\Psi }}}=\varepsilon 2\pi {\int }_{0}^{2\pi }{c}^{\ast }(\alpha ,t)g(\alpha )d\alpha $$

Substituting these relations into (10) and separating the Fourier modes, we get, to the first order in *ε*, the following equation for the evolution of *c*:

13 $$\frac{\partial c(\alpha ,t)}{\partial t}=\frac{1}{2}[\lambda {e}^{-i{{\rm{\Theta }}}_{1}}{\int }_{0}^{2\pi }c(x,t)g(x)dx+{e}^{-i({{\rm{\Theta }}}_{2}+\alpha )}{\int }_{0}^{2\pi }c(x,t)g(x)dx].$$

With the exponential in time ansatz $c(\alpha ,t)={e}^{\gamma t}b(\alpha )$ Eq. (13) takes the form

14 $$\gamma b(\alpha )=\frac{1}{2}[\lambda {e}^{-i{{\rm{\Theta }}}_{1}}{\int }_{0}^{2\pi }b(x)g(x)dx+{e}^{-i({{\rm{\Theta }}}_{2}+\alpha )}{\int }_{0}^{2\pi }b(x)g(x)dx]$$

Eq. (14) can be solved in a self-consistent way. We define $A=\frac{1}{2}{\int }_{0}^{2\pi }b(x)g(x)dx$, so that $b(\alpha )$ can be expressed as $b(\alpha )=\frac{1}{2\gamma }[\lambda {e}^{-i{{\rm{\Theta }}}_{1}}A+{e}^{-i({{\rm{\Theta }}}_{2}+\alpha )}A]$. Thus we obtain from Eq. (14)

15 $$1=\frac{\lambda {e}^{-i{{\rm{\Theta }}}_{1}}}{4\gamma }+\frac{{e}^{-i{{\rm{\Theta }}}_{2}}}{4\gamma }{\int }_{0}2\pi {e}^{-i\alpha }g(\alpha )d\alpha $$

For a particular case of the von Mises distribution (7) the integral can be explicitly calculated and we obtain

$$\gamma =\frac{\lambda {e}^{-i{{\rm{\Theta }}}_{1}}}{4}+\frac{{e}^{-i{{\rm{\Theta }}}_{2}}{I}_{1}({\rm{\Delta }})}{4{I}_{0}({\rm{\Delta }})}$$

Stability is determined by the real part of *γ*, so the critical values of the parameters, separating stable and unstable incoherent state, can be obtained from the equation

16 $${\rm{Re}}(\gamma )=\frac{\lambda {\cos {\rm{\Theta }}}_{1}}{4}+\frac{{I}_{1}({\rm{\Delta }}){\cos {\rm{\Theta }}}_{2}}{4{I}_{0}({\rm{\Delta }})}=0$$

### Self-consistent approach

We seek for partially synchronous solutions in the model described by Eq. (6) in the form of uniformly rotating states, with some frequency Ω, to be defined in the procedure. In terms of the distribution density $\rho (\phi ,\alpha ,t)$ these solutions are traveling waves. It is convenient to transform all the variables to the rotating frame, where we then will look for stationary solutions of the distribution of the phases. Furthermore, because we treat the problem in the thermodynamic limit, it is suitable to parametrize the phases by the value of parameter *α* (we will write it as a lower index for the phase variables). We thus introduce new phases

$$\psi =\theta -{\rm{\Psi }}-{{\rm{\Theta }}}_{1}$$

and assume that $\dot{{\rm{\Psi }}}={\rm{\Omega }}$. In these variables the ensemble driven by two mean fields (8) can be written as

17 $${\dot{\psi }}_{\alpha }=-\,{\rm{\Omega }}-R\lambda \,\sin \,{\psi }_{\alpha }-R\,\sin ({\psi }_{\alpha }+\beta -\alpha ),R{e}^{-i{{\rm{\Theta }}}_{1}}=\langle {e}^{i\psi }\rangle ={\int }_{0}^{2\pi }d\alpha g(\alpha )\exp [i{\psi }_{\alpha }]$$

where $\beta ={\Theta }_{1}-{\Theta }_{2}$. It is convenient to introduce auxiliary parameters *a*, *r*, s according to

18 $$a=\frac{R}{{\rm{\Omega }}},{r}^{2}={a}^{2}{\lambda }^{2}+2{a}^{2}\lambda \,\cos (-\beta +\alpha )+{a}^{2},s={\rm{atan2}}(a\,\sin (-\beta +\alpha ),a\lambda +a\,\cos (-\beta +\alpha \mathrm{))}.$$

Then

19 $${\dot{\psi }}_{\alpha }={\rm{\Omega }}(-1-r\,\sin ({\psi }_{\alpha }-s))$$

One can see that the essential parameters for the dynamics of oscillators in (19) are *r* and *s*, which depend explicitly on *a*, *λ*, *β*, *α*. This suggests the following strategy to find the solutions of the model:

1. For fixed parameters *a*, *λ*, *β*, *α* one finds a stationary density of phases $w({\psi }_{\alpha }|a,\lambda ,\beta ,\alpha )$ and the corresponding average
  20 $$z(a,\lambda ,\beta ,\alpha )=\langle {e}^{i{\psi }_{\alpha }}\rangle ={\int }_{0}^{2\pi }d{\psi }_{\alpha }\exp [i{\psi }_{\alpha }]w({\psi }_{\alpha }|a,\lambda ,\beta ,\alpha )$$
2. After that one uses the value of *z* to find the average over the distribution of *α*
  $$\langle {e}^{i\psi }\rangle ={\int }_{0}^{2\pi }d\alpha g(\alpha )z(a,\lambda ,\beta ,\alpha )=F(a,\lambda ,\beta )\exp [iQ(a,\lambda ,\beta )].$$
3. Applying (17) and (18) one then obtains the parameters ${{\rm{\Theta }}}_{\mathrm{1,2}}$ as well as the main characterizations of the dynamics, the order parameter *R* and the frequency Ω, in a parametric form as functions of the introduced auxiliary parameters *a*, *β*:

21 $$\begin{array}{ll}R=F(a,\lambda ,\beta ), & {{\rm{\Theta }}}_{1}=-\,Q(a,\lambda ,\beta ),\\ {{\rm{\Theta }}}_{2}=-\,Q(a,\lambda ,\beta )-\beta , & {\rm{\Omega }}=\frac{F(a,\lambda ,\beta )}{a}.\end{array}$$

We now present the steps in this procedure in details. The first goal is to find a distribution of the phases governed by Eq. (19). There are two types of possible regimes for the phase: the locked state for *r* > 1 and the rotating state for *r* < 1. The locked state has a definite value of the phase $\psi ={\psi }_{0}$, thus the distribution is the delta-function and ${z}_{l}=\exp [i{\psi }_{0}]$ (here index *l* denotes locked states). We have to choose the stable locked state, therefore the value of ${\psi }_{0}$ depends also on the sign of Ω, i.e. on the sign of parameter *a*:

22 $${z}_{l}=(\begin{array}{cc}{e}^{is}(\sqrt{1-{\mathrm{(1/}r)}^{2}}-i/r) & {\rm{for}}\,{\rm{\Omega }} > 0\\ {e}^{is}(-\sqrt{1-{\mathrm{(1/}r)}^{2}}-i/r) & {\rm{for}}\,{\rm{\Omega }} < 0\end{array}$$

For *r* < 1 there are no locked states and the phases rotate. Here the stationary distribution is just inverse proportional to the velocity

$$w(\psi )=\frac{C}{|\dot{\psi }|}=\frac{C}{1+r\,\sin (\psi -s)}$$

where *C* is the normalization constant. Using standard integrals we get

23 $$w(\psi )=\frac{\sqrt{1-{r}^{2}}}{2\pi \mathrm{(1}+r\,\sin (\psi -s))},{z}_{r}=i{e}^{is}\frac{-1+\sqrt{1-{r}^{2}}}{r}$$

(Here index *r* denotes rotating states). Combining Eqs (22), (23) we get the final expression for the parameters *F*, *Q*:

24 $$\langle {e}^{i\psi }\rangle =F{e}^{iQ}={\int }_{0}^{2\pi }d\alpha {z}_{l}g(\alpha )+{\int }_{0}^{2\pi }d\alpha {z}_{r}g(\alpha ),g(\alpha )=\frac{{e}^{{\rm{\Delta }}\cos \alpha }}{2\pi {I}_{0}({\rm{\Delta }})}$$

Additionally, we can calculate the portion of the locked oscillators *P*_*l*_:

25 $${P}_{l}(a,\lambda ,\beta )={\int }_{0}^{2\pi }d\alpha H(\alpha )g(\alpha )$$

where

$$H(\alpha )=\{\begin{array}{cc}1 & {\rm{if}}\,r > \mathrm{1,}\\ 0 & {\rm{if}}\,r < \mathrm{1,}\end{array}$$

is the indicator function for the locked states. We call *P*_*l*_ the locking parameter. In the standard Kuramoto model, *P*_*l*_ is proportional to the mean field amplitude, but in our case we will see a nontrivial behavior of *P*_*l*_: in some cases all the oscillators are locked, i.e. *P*_*l*_ = 1, while the order parameter *R* is rather small.

### Reformulation in terms of Ott-Antonsen equations

Generally, to study stability of the stationary rotating solutions described above, we need to analyze generic perturbations of the equation for the density (10). We however restrict ourselves to a class of perturbations lying on the so-called Ott-Antonsen manifold^26^. The coupling in our model has a pure sin form, therefore the Ott-Antonsen (OA) ansatz leading to an integral equation for the local order parameter $z(\alpha ,t)=\langle {e}^{i{\psi }_{\alpha }}\rangle$ is possible. In this Ansatz one represents the density as $\rho (\psi ,\alpha ,t)={\mathrm{(2}\pi )}^{-1}[1+{\sum }_{m}({z}^{m}{e}^{-im\psi }+{({z}^{\ast })}^{m}{e}^{im\psi })]$. Then one applies this to Eq. (17) and obtains (see refs 26 and 36 for details)

26 $$\begin{array}{rcl}\dot{z}(\alpha ,t) & = & -i{\rm{\Omega }}z+\frac{1}{2}((Z\lambda {e}^{i{{\rm{\Theta }}}_{1}}+Z{e}^{i{{\rm{\Theta }}}_{2}}{e}^{i\alpha })-({Z}^{\ast }\lambda {e}^{-i{{\rm{\Theta }}}_{1}}+{Z}^{\ast }{e}^{-i{{\rm{\Theta }}}_{2}}{e}^{-i\alpha }){z}^{2}(\alpha )),\\ Z & = & {\int }_{0}^{2\pi }g(\alpha )z(\alpha \mathrm{)}.\end{array}$$

Because the found solutions are steady states of system (26), the linearization is straightforward. Furthermore, we discretize the resulting linear integral equations using a discrete (size *L*) representation of the parameter *α* on the circle: ${\alpha }_{k}={\alpha }_{0}+2\pi k/L$, $k=0,\ldots ,L-1$. Here the offset $0\le {\alpha }_{0} < 2\pi /L$ is a free parameter. It can be used to distinguish the continuous and the discrete parts of the spectrum (cf. ref. 37).

### Solutions with a vanishing order parameter

Here we analyze the limit *R* → 0 of the constructed above traveling wave solutions. Because, according to (21), *R* = Ω*a*, there are two possibilities for order parameter *R* to vanish: *a* = 0 and Ω = 0.

1. Case *a* → 0.
  For *a* → 0 we have also *r* → 0. Eq. (23) in the limit *r* → 0 reduces to
  $${\langle {e}^{i\psi }\rangle }_{r}=i{e}^{is}\frac{-1+\sqrt{1-{r}^{2}}}{r}\approx -\frac{r}{2}i{e}^{is}$$
  We use
  $$r{e}^{is}=a\lambda +a\,\cos (\alpha -\beta )+ia\,\sin (\alpha -\beta )$$
  and get from (24)
  27 $$\begin{array}{rcl}F{e}^{iQ} & = & -\frac{i}{2}{\int }_{0}^{2\pi }d\alpha g(\alpha )[a\lambda +a\,\cos (\alpha -\beta )+ia\,\sin (\alpha -\beta )]\\  & = & -\,\frac{i}{2}[\lambda a+a{e}^{-i\beta }{\int }_{0}^{2\pi }d\alpha g(\alpha ){e}^{i\alpha }]=-\frac{i}{2}[\lambda a+a{e}^{-i\beta }\frac{{I}_{1}({\rm{\Delta }})}{{I}_{0}({\rm{\Delta }})}]\end{array}$$
  We substitute here $F=R={\rm{\Omega }}a$, $Q=-\,{{\rm{\Theta }}}_{1}$ and $\beta ={{\rm{\Theta }}}_{1}-{{\rm{\Theta }}}_{2}$ and obtain in the limit *a* → 0
  $$2{\rm{\Omega }}=-\,[\lambda (i\,\cos \,{{\rm{\Theta }}}_{1}-\,\sin \,{{\rm{\Theta }}}_{2})+(i\,\cos \,{{\rm{\Theta }}}_{2}-\,\sin \,{{\rm{\Theta }}}_{2})\frac{{I}_{1}({\rm{\Delta }})}{{I}_{0}({\rm{\Delta }})}]$$
  Because Ω is real, the condition
  $$\lambda \,{\cos {\rm{\Theta }}}_{1}+{\cos {\rm{\Theta }}}_{2}\frac{{I}_{1}({\rm{\Delta }})}{{I}_{0}({\rm{\Delta }})}=0$$
  should be fulfilled. This is exactly the condition for the linear stability border (16).
2. Case $${\rm{\Omega }}=0$$.

In this case $F=0$ and according to (24)

28 $$0={\int }_{0}^{2\pi }d\alpha {\langle {e}^{i\psi }\rangle }_{r,l}g(\alpha )$$

This relation defines two real equations, i.e. two constrains on parameters *a*, *β*, *λ*. This means that there may be a curve (or a set of curves) *β*(*λ*) for which condition (28) is fulfilled, so that solutions with vanishing order parameter *R* = 0 exist on this set. This is exactly the curve of anomalous solutions with vanishing *R* presented in Fig. 1.

## Electronic supplementary material

Supplementary Information
