# Supplementary material for: Dynamics of oscillators globally coupled via two mean fields
Source: Sci Rep. 2017 May 18;7:2104. doi: 10.1038/s41598-017-02283-1 (PMC5437098; doi:10.1038/s41598-017-02283-1)
Supplement: Supplementary file 1 — Supplementary Information [file 41598_2017_2283_MOESM1_ESM.pdf]

Dynamics of oscillators globally coupled via two mean fields

Supplementary Information

Xiyun Zhang,[1,2] Arkady Pikovsky,[2,3] and Zonghua Liu[1]

[1]*Department of Physics, East China Normal University, Shanghai, 200062, P. R. China*

[2]*Institute for Physics and Astronomy, University of Potsdam, Karl-Liebknecht-Str. 24/25, 14476 Potsdam-Golm, Germany*

[3]*Department of Control Theory, Nizhny Novgorod State University, Gagarin Av. 23, 606950, Nizhny Novgorod, Russia*

(Dated: March 16, 2017)

SUPPLEMENTARY FIGURES

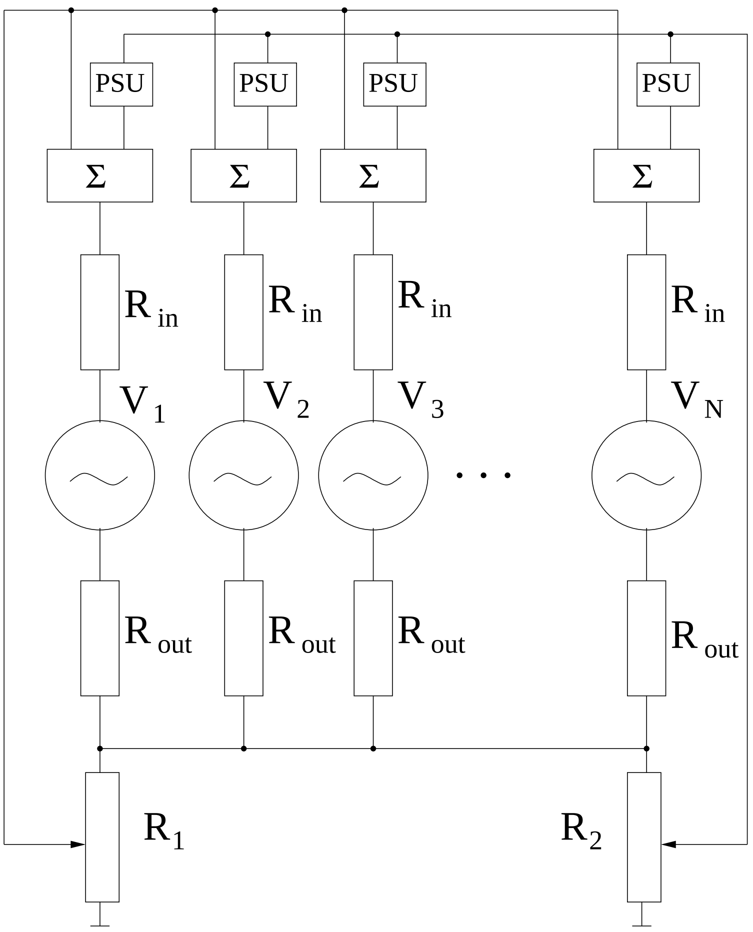

FIG. 1. **A possible electronic circuit implementing two mean field coupling.** It consists of N possibly identical (within possible accuracy of realization) Wien-bridge selv-sustained oscillators. Their equations are equivalent to the van der Pol equation. Two global couplings are organized via the common resistive loads R_1 and R_2. The voltages from these loads are fed back to all oscillators. One voltage is fed back directly, while the other voltage is fed back via phase shifts units. Thus, this scheme implements the basic model studied in this paper.

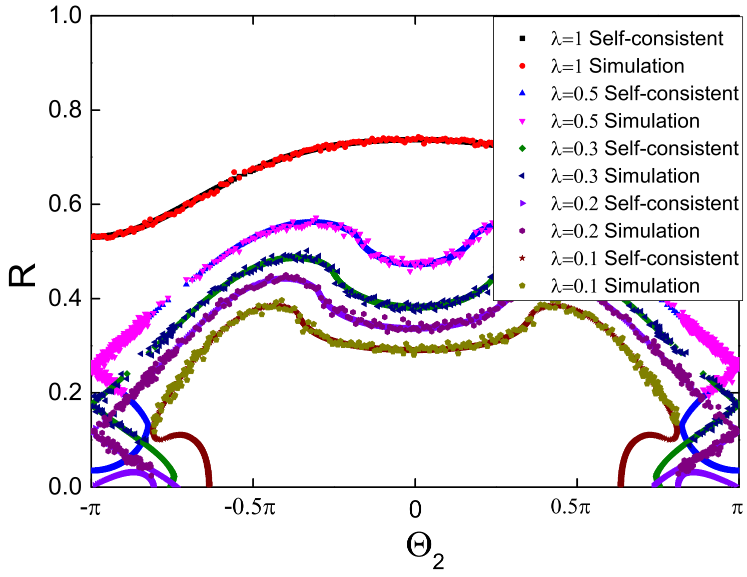

FIG. 2. **Compare the self-consistent solution and direct simulation results**, the parameters are the same with Fig. 3 in the main body.

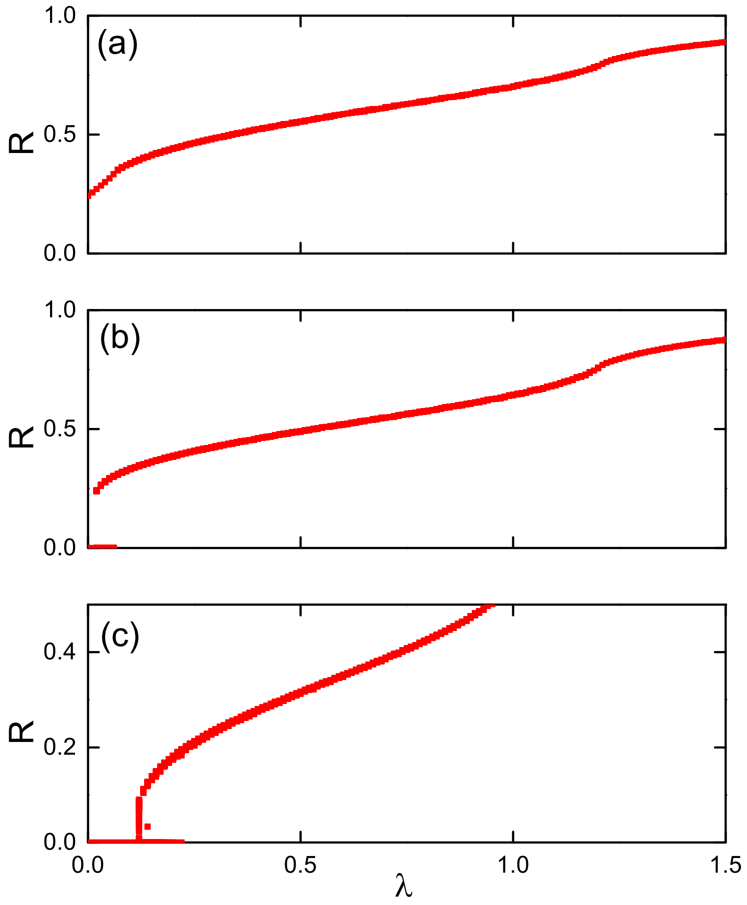

FIG. 3. **The global order parameter of traveling wave states for $\Theta_1 = 0$ and $\Delta = 0.5$, as functions of λ.** The values of Θ_2 are (a): $\Theta_2 = -0.4\pi$; (b): $\Theta_2 = -0.6\pi$; and (c): $\Theta_2 = -0.9\pi$. Results are from simulations with a N=100 system and for each λ, we plot the final order parameter generated from 1500 random initial conditions.

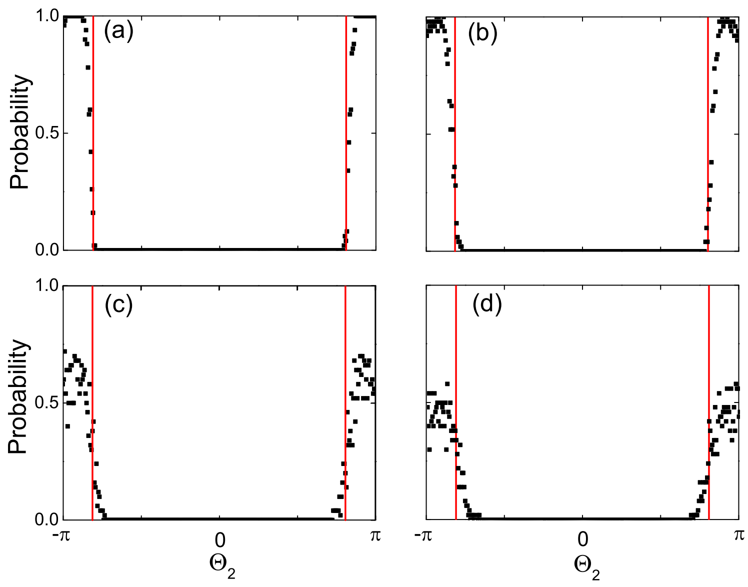

FIG. 4. **Probability of the system ending up with incoherent state in 50 realizations with random initial conditions.** Parameters are $\Theta_1 = 0$, $\Delta = 0.5$ and $\lambda = 0.2$. The system sizes are (a): N=10000; (b): N=5000, (c): N=1000 and (d): N=500. Red lines show the linear stability boundaries according to Eq. (15)
